# The prevalence and management of chronic pain in the Chinese population: findings from the China Pain Health Index (2020)

**DOI:** 10.1186/s12963-022-00297-0

**Published:** 2022-11-04

**Authors:** Yingying Jiang, Tingling Xu, Fan Mao, Yu Miao, Botao Liu, Liyuan Xu, Lingni Li, Nikoletta Sternbach, Maigeng Zhou, Bifa Fan

**Affiliations:** 1 National Center for Chronic and Non-Communicable Disease Control and Prevention, Chinese Center for Disease Control and Prevention, Beijing, China; 2 China-Japan Friendship Hospital, Beijing, China; 3 Kantar China, Shanghai, China; 4 Kantar US, New York, USA

**Keywords:** China Pain Health Index study (CPHI), Evaluation, Chronic pain, Population

## Abstract

**Background:**

Chronic pain is a common disease; about 20% of people worldwide suffer from it. While compared with the research on the prevalence and management of chronic pain in developed countries, there is a relative lack of research in this field in China. This research aims to construct the China Pain Health Index (CPHI) to evaluate the current status of the prevalence and management of chronic pain in the Chinese population.

**Methods:**

The dimensions and indicators of CPHI were determined through literature review, Delphi method, and analytical hierarchy process model, and the original values ​​of relevant indicators were obtained by collecting multi-source data. National and sub-provincial scores of CPHI (2020) were calculated by co-directional transformation, standardization, percentage transformation of the aggregate, and weighted summation.

**Results:**

The highest CPHI score in 2020 is Beijing, and the lowest is Tibet. The top five provinces are Beijing (67.64 points), Shanghai (67.04 points), Zhejiang (65.74 points), Shandong (61.16 points), and Tianjin (59.99 points). The last five provinces are Tibet (33.10 points), Ningxia (37.24 points), Guizhou (39.85 points), Xinjiang (39.92 points), and Hainan (40.38 points). The prevalence of chronic pain is severe in Heilongjiang, Chongqing, Guizhou, Sichuan, and Fujian. Guizhou, Hainan, Xinjiang, Beijing, and Guangdong display a high burden of chronic pain. The five provinces of Guangdong, Shanghai, Beijing, Jiangsu, and Zhejiang have better treatment for chronic pain, while Tibet, Qinghai, Jilin, Ningxia, and Xinjiang have a lower quality of treatment. Beijing, Shanghai, Qinghai, Guangxi, and Hunan have relatively good development of chronic pain disciplines, while Tibet, Sichuan, Inner Mongolia, Hebei, and Guizhou are relatively poor.

**Conclusion:**

The economically developed provinces in China have higher CPHI scores, while economically underdeveloped areas have lower scores. The current pain diagnosis and treatment situation in economically developed regions is relatively good, while that in financially underdeveloped areas is rather poor. According to the variations in the prevalence and management of chronic pain among populations in different provinces in China, it is necessary to implement chronic pain intervention measures adapted to local conditions.

## Background

Chronic pain lasts longer than 3 to 6 months despite medication or treatment. Chronic pain is a common disease; about 20% of people worldwide suffer from it [1]. The 2016 Global Burden of Disease Study reported that the prominence of pain and pain-related diseases is the leading cause of disability and disease burden worldwide. Globally, the burden of chronic pain is escalating that 1.9 billion people were affected by recurrent tension-type headache, the most common chronic symptom. Low back and neck pain remain the leading cause of disability internationally, with other chronic pain conditions prominently featured in the top 10 disabilities [2].

Studies have shown that in middle-income and low-income countries, the prevalence of chronic pain in adults is 33%, and it is as high as 56% in the elderly [3]. There is a difference in the prevalence of chronic pain among different groups. Situations such as the increasing burden of pain, the rising medical costs caused by lack or low-quality pain management, and the physical, psychological, and economic losses caused by it to individuals and their family members have made chronic pain an important public health problem.

Data show that the prevalence rate of chronic pain in the Chinese population exceeds 30% [4]. Historically, China is a country that treats pain as a disease. However, the pain relief effect in the Chinese population is currently poor. Possible barriers include cultural and philosophical differences between China and the West, patient misunderstandings about pain management, fear of medicine use, and lack of professional knowledge about pain management [4]. Moreover, compared with the research on the prevalence and management of chronic pain in developed countries, there is a relative lack of research in this field in China [5]. The maintenance of the well-being of the chronic pain population depends on measures such as improving patients’ awareness and attendance rate, improving the standardization of physician’s diagnosis and treatment, and developing the construction of pain disciplines. However, we are not clear about the situation of chronic pain in China and the specific conditions of each province. Therefore, National Center for Chronic and Non-communicable Disease Control and Prevention, the Chinese Center for Disease Control and Prevention (China CDC), and the China–Japan Friendship Hospital jointly launched the China Pain Health Index study (CPHI) [6]. This study adopted a comprehensive evaluation method to determine a set of index systems to evaluate the prevalence, prevention, and control of chronic pain in the population in China. Each index was assigned with weight, and the score of CPHI was calculated through the aggregation of each index. This article will analyze the current status of chronic pain prevalence and management in China based on the results of CPHI (2020).

## Method

### Index system and data sources of China Pain Health Index

First, the dimensions and indicators of CPHI are determined through literature review, the Delphi method, and the analytical hierarchy process (AHP) model. CPHI (2020) includes 16 indicators in 4 dimensions. The four dimensions are the prevalence of pain disease, the burden of pain disease, diagnosis and treatment, and the construction of pain disease disciplines. The corresponding weights are 0.1680, 0.1922, 0.3350, and 0.3048. Data sources for the 16 indicators include China’s provincial burden of disease research (GBD China) [7], the National Health and Wellness Survey (NHWS) database [8], registration data of the Chinese Medical Doctor Association (CMDA) [9], and self-reported data of doctors’ surveys. The GBD China study is part of the Global Burden of Disease Study, which covers the world and assesses the mortality and disability caused by major diseases, injuries, and risk factors. China NHWS is a cross-sectional, Internet-based survey of urban adults (age >  = 18) that provides a unique look into the healthcare market from the consumer’s viewpoint. CDMA is a national, hospital-based, non-profit organization formed voluntarily by practicing physicians and assistant physicians. The specific definitions and data sources of each indicator in the four dimensions of CPHI are shown in Table 1.

**Table 1:** Index definitions and data sources of CPHI (2020)

Dimension	No	Indicator	Definition	Data source
A Prevalence of chronic pain	A01	Headache prevalence	The proportion of the surveyed population who has been diagnosed with headache after age standardization	GBD China(2019)
	A02	Musculoskeletal pain prevalence	The proportion of people who have been diagnosed with musculoskeletal pain after age standardization	GBD China(2019)
	A03	Headache incidence rate	Among the surveyed population, the proportion of newly diagnosed headaches each year after age standardization	GBD China(2019)
	A04	Incidence rate of musculoskeletal pain	Among the surveyed population, the proportion of newly diagnosed musculoskeletal pain each year after age standardization	GBD China(2019)
B Disease burden of chronic pain	B01	Headache DALYs	The number of years lost due to ill-health, disability, or early death caused by headaches including YLLs and YLDs	GBD China(2019)
	B02	Musculoskeletal pain DALYs	The number of years lost due to ill-health, disability or early death caused by musculoskeletal pain including YLLs and YLDs	GBD China(2019)
	B03	Direct economic burden of pain	The per capita annual medical expenses including all related medical expenses such as drugs, surgery, medical treatment, and examination incurred by pain patients due to the treatment of pain	NHWS
	B04	Indirect economic burden of pain	The economic loss of pain patients to the patient himself and to the society including the loss of lost work or absenteeism	NHWS
C Treatment of chronic pain	C01	Standardization of doctors’ behavior in diagnosis and treatment	Average score on the Doctor’s Standardization Questionnaire for Diagnosis and Treatment	doctors’ survey
	C02	Visiting rate of pain patients	Proportion of patients who visited the hospital in the past month reported pain	NHWS
	C03	Treatment rate of pain patients	The proportion of pain patients receiving treatment (including drugs, surgery, physical therapy, auxiliary treatment, psychotherapy, etc.)	NHWS
	C04	Satisfaction of pain patients with analgesic medication	The proportion of pain patients who are satisfied after receiving analgesic medication	NHWS
D development of chronic pain disciplines	D01	Pain department coverage	The number of secondary and tertiary hospitals with pain departments accounted for the proportion of all secondary and tertiary hospitals	CMDA
	D02	Pain physicians per million population	The number of pain physicians per million population	CMDA
	D03	Average hours of pain training per medical staff per year	Among the survey respondents, the average number of hours per person receives continuing education on pain per year	doctors’ survey
	D04	The current academic qualifications of pain physicians	Among the survey respondents, the proportion of pain physicians with master’s degree or above	CMDA

Headache disorders include Migraine and Tension-type headache. Musculoskeletal disorders include Rheumatoid arthritis, Osteoarthritis, Low back pain, Neck pain, Gout and other musculoskeletal disorders

### Index calculation

Due to the differences in the dimensions, magnitudes, and content of the indicators, the calculation of the CPHI (2020) involves several major steps such as co-directional transformation, standardization, percentage transformation of the aggregate, and weighted summation.

#### Co-directional transformation

The full point of CPHI is 100, and the higher the score, the better the pain prevention and control work is. The purpose of adopting the exact trend conversion for each index is to make the index reflecting the health problem consistent with the CPHI reflecting the health status. That is, through the co-directional transformation, the higher the value of each index reflects the better health status, the better the current situation of pain diagnosis and treatment and the construction of pain disciplines. For example, indicators with a higher value representing a worse health status, such as prevalence and incidence, multiplied the original value by − 1, so the higher the value represents, the better the health status. For other indicators such as satisfaction and the number of pain physicians, the higher the value represents, the better the health status, diagnosis and treatment, and the development of disciplines, so there is no need to perform that. In this index, eight indicators, including A01–A04 and B01–B04, multiplied the original value by − 1 for co-directional transformation.

#### Standardization

A preliminary analysis showed that most indicators were subject to normal distribution. Therefore, the standard normal transformation was used to remove the dimension of each index so that all transformed indexes obey the standard normal distribution with a mean value of 0 and a standard deviation of 1. The transformation formula was (see formula 1):

1 $$z_{i} = \frac{{X_{i} - \mu_{i} }}{{\sigma_{i} }}$$

${z}_{i}$ refers to the mark of ith index after standard normal transformation, ${X}_{i}$ is the initial or co-directional value of the ith index, ${\mu }_{i}$ is the mean value of ith index of each China province, and ${\sigma }_{i}$ refers to the standard deviation (SD) of ith index of each province.

#### Percentage transformation

To ensure that the final value fell between 0 and 100, it was necessary to undertake percentage transformations. This was done by calculating the area under the standard normal distribution curve on the left side of value *z*. As for any standard normalized index ${z}_{i}$, its score was ${S}_{i}$ (see formula 2):

2 $$S_{i} = 100 \cdot \int\limits_{ - \infty }^{{Z_{i} }} {\frac{1}{{\sqrt {2\pi } }}e^{{ - \frac{{x^{2} }}{2}}} {\text{d}}x}$$

#### Weighted summation

Combining each index’s standard normalized mark and weight, we calculated the marks of different dimensions in each province and final CPHI scores. The calculation method was (see formula 3):

3 $${\text{CPHI}} = \sum\limits_{i = 1}^{n} {S_{i} \cdot w_{i} }$$

*n* is the number of indices of a certain dimension or the number of all indices, ${S}_{i}$ is the marks of standard normalization, and ${w}_{i}$ is the weight of the indicator. The CPHI (2020) has a full score of 100. The higher the score, the better the pain health in the region.

## Results

### CPHI scores and province rankings

**Fig. 1:**
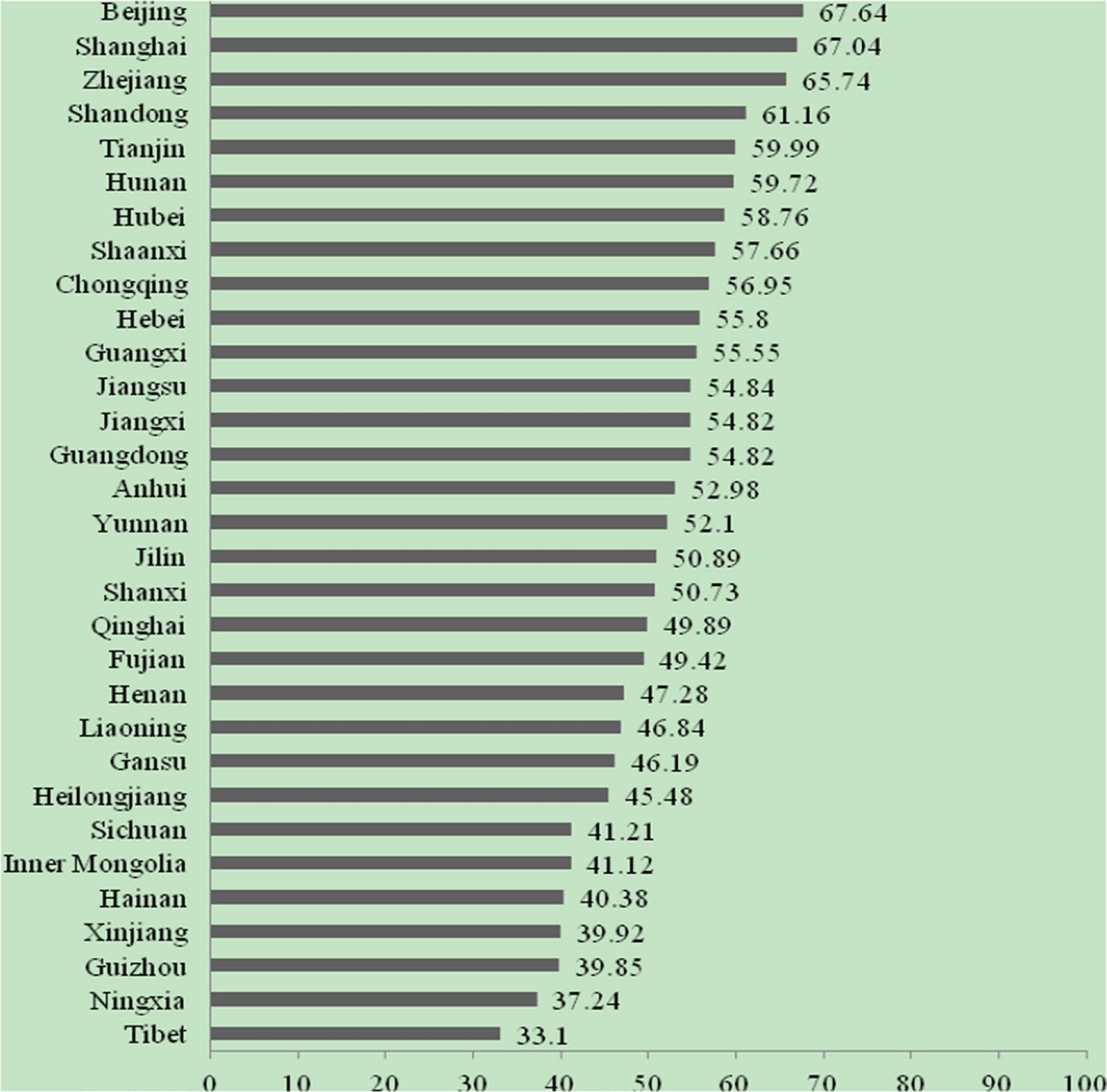
Provincial scores of China Pain Health Index (2020)

Beijing has the highest CPHI score in China in 2020, and Tibet has the lowest score. The top five scoring provinces are Beijing (67.64 points), Shanghai (67.04 points), Zhejiang (65.74 points), Shandong (61.16 points), and Tianjin (59.99 points). The bottom five provinces are Tibet (33.10 points), Ningxia (37.24 points), Guizhou (39.85 points), Xinjiang (39.92 points), and Hainan (40.38 points). See Fig. 1.

### CPHI scores in different dimensions

**Fig. 2:**
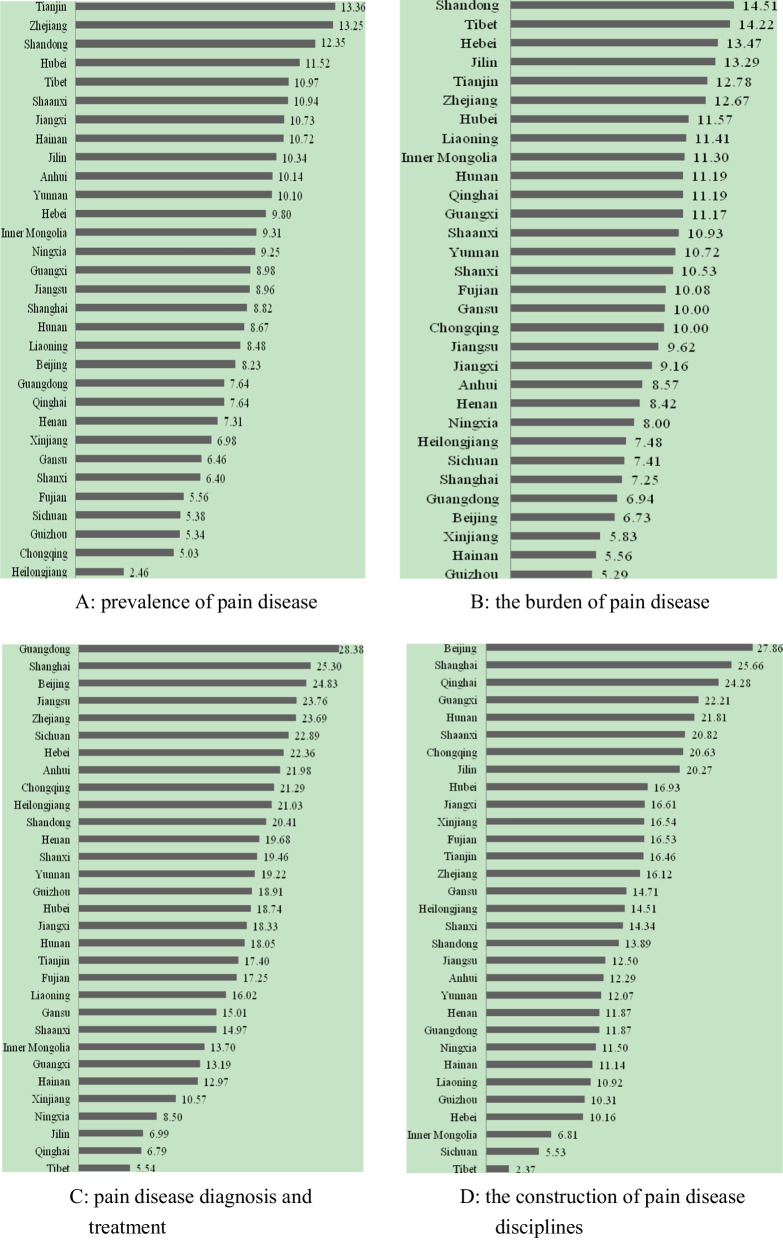
Provincial scores of China Pain Health Index (2020) by dimensions. **a** Dimension A: prevalence of pain disease. **b** Dimension B: the burden of pain disease. **c** Dimension C: pain disease diagnosis and treatment. **d** Dimension D: the construction of pain disease disciplines

The prevalence of chronic pain is more severe in Heilongjiang (2.46), Chongqing (5.03), Guizhou (5.34), Sichuan (5.38), and Fujian (5.56). The disease burden of chronic pain in Guizhou (5.29), Hainan (5.56), Xinjiang (5.83), Beijing (6.73), and Guangdong (6.94) is relatively severe. The five provinces of Guangdong (28.38), Shanghai (25.30), Beijing (24.83), Jiangsu (23.76), and Zhejiang (23.69) had better treatment of chronic pain, while the five provinces of Tibet (5.54), Qinghai (6.79), Jilin (6.99), Ningxia (8.50), and Xinjiang (10.57) had poor treatment. The five provinces of Beijing (27.86), Shanghai (25.66), Qinghai (24.28), Guangxi (22.21), and Hunan (21.81) have relatively good development of chronic pain disciplines, while the five provinces of Tibet (2.37), Sichuan (5.53), Inner Mongolia (6.81), Hebei (10.16), and Guizhou (10.31) are relatively poor. See Fig. 2.

### Original value by province

#### Chronic pain prevalence and disease burden in the Chinese population

**Fig. 3:**
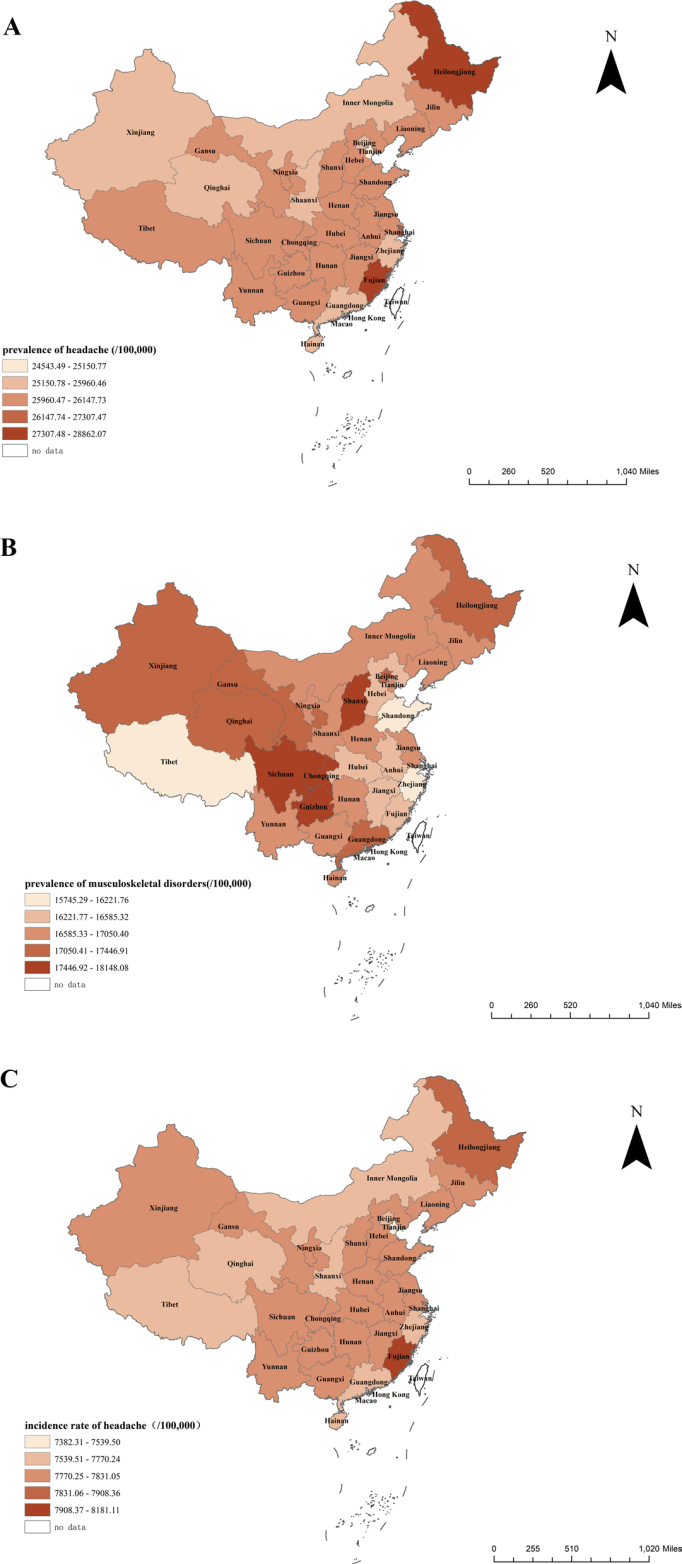

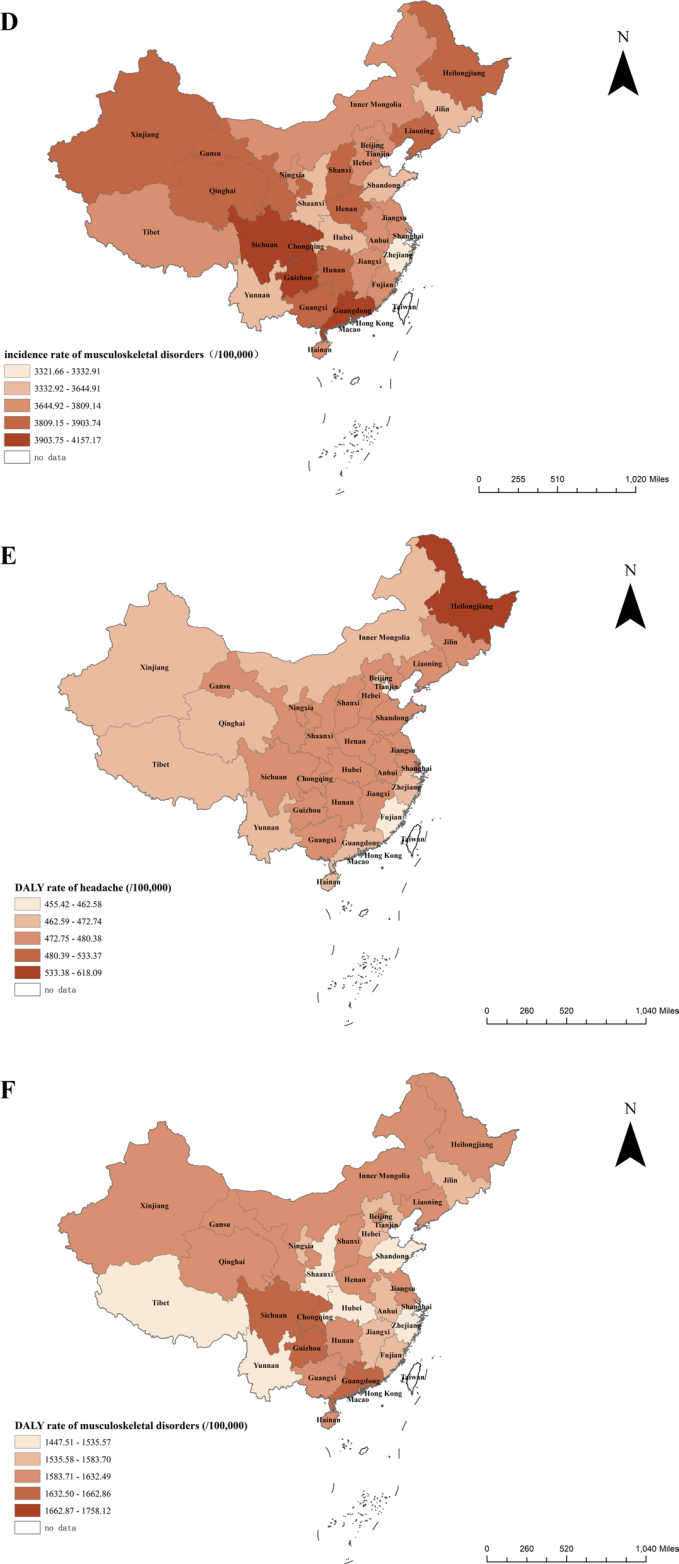
Prevalence of pain and disease burden in various provinces in China. **a** The prevalence of headache at the provincial level in China. **b** The prevalence of musculoskeletal disorders at the provincial level in China. **c** The incidence rate of headache at the provincial level in China. **d** The incidence rate of musculoskeletal disorders at the provincial level in China. **e** The DALY rate of headache at the provincial level in China. **f** The DALY rate of musculoskeletal disorders at the provincial level in China

The prevalence of headaches in Heilongjiang, Fujian, and Shanghai is relatively high, achieving 28.86%, 28.68%, and 27.31%, respectively. The prevalence of skeletal muscle pain in Chongqing, Sichuan, and Guizhou is18.03%, 17.89%, and 17.86%, respectively, which is relatively high in China. In terms of the incidence of headache, Heilongjiang, Fujian, and Shanghai have relatively high values, which are 8.18%, 7.91%, and 7.83%, respectively. The incidence of skeletal muscle pain in Guangdong, Guizhou, Sichuan, and Chongqing is relatively high, with 3964.7 per 100,000, 3960.9 per 100,000, 3959.3 per 100,000, and 3950.9 per 100,000, respectively. The DALYs rate of headaches in Heilongjiang and Shanghai was higher, reaching 618.1/100,000 and 533.4/100,000, respectively. The DALYs rates of skeletal muscle pain in Chongqing, Guangdong, and Guizhou are higher, reaching 1661.7/100,000, 1656.6/100,000, and 1653.6/100,000, respectively. Figure 3 shows the prevalence and disease burden of pain in various provinces in China.

#### Chronic pain management in the Chinese population

The outpatient visiting rate for pain patients in China is 68.2%, the treatment rate is 77.8%, and the satisfaction rate of analgesic use is 64.3%. Gansu, Guizhou, and Hainan have a higher rate of outpatient visiting rate, exceeding 90%; Qinghai has the lowest visiting rate, only 58.2%. The three provinces of Fujian, Zhejiang, and Heilongjiang have a higher treatment rate for pain patients, exceeding 85%; Qinghai, Hainan, and Jilin have the lowest treatment rate for pain patients, at 36.1%, 51.2%, and 52.8%, respectively. The satisfaction of pain patients in Henan, Guangdong, Shanghai, Hebei, and Heilongjiang with analgesic drug treatment was higher, which was 71.3%, 70.0%, 69.9%, 69.5%, and 69.1%, respectively; Ningxia, Hainan, Gansu, Qinghai, Tibet, Xinjiang, Inner Mongolia, and Guizhou have lower satisfaction levels, all of which are less than 50%. See Table 2.

**Table 2:** Visiting rate, treatment rate, and satisfaction rate of patients with pain (%)

Province name	C02 Pain patient visiting rate	C03 Treatment rate of pain patients	C04 satisfaction of pain patients with analgesic medication
Gansu	93.2	67.3	36.5
Guizhou	90.2	77.5	49.3
Hainan	90.1	51.2	35.1
Shanghai	85.9	79.0	69.9
Anhui	85.9	74.3	54.1
Guangdong	85.4	79.7	70.0
Beijing	84.6	80.7	66.2
Jiangsu	84.5	72.6	61.3
Shaanxi	84.5	82.5	52.4
Henan	84.3	72.5	71.3
Sichuan	83.8	77.9	62.9
Zhejiang	83.7	86.3	55.0
Hubei	82.4	75.6	52.9
Shanxi	81.3	62.3	63.8
Tianjin	81.1	81.2	65.2
Liaoning	80.5	72.4	61.7
Fujian	79.9	89.7	57.5
Hebei	79.2	78.0	69.5
Chongqing	75.6	70.2	65.6
Jilin	75.4	52.8	59.6
Yunnan	75.3	79.8	59.0
Jiangxi	75.0	62.3	58.9
Heilongjiang	74.7	85.3	69.1
Inner Mongolia	70.6	75.3	45.8
Hunan	69.9	62.1	58.3
Guangxi	69.3	66.5	65.3
Shandong	68.6	75.2	65.6
Tibet	68.3	60.9	40.9
Xinjiang	66.5	73.8	42.3
Ningxia	62.9	69.6	29.9
Qinghai	58.2	36.1	40.9

Shanghai, Beijing, and Guangxi provinces have higher coverage rates of pain departments, 39.7%, 36.0%, and Guangxi, 33.7%, respectively. Inner Mongolia, Tibet, and Sichuan provinces have lower pain coverage rates, at 5.3%, 7.6%, and 9.4%, respectively. Qinghai, Beijing, and Shaanxi provinces have the most significant number of pain doctors per million, 19.1, 15.7, and 15.6, respectively. The four provinces of Inner Mongolia, Tibet, Sichuan, and Guangdong have the least number of pain doctors, with less than 5 per million population. Shanghai has the highest proportion of postgraduates of pain physicians with a master’s degree or above, at 52.0%. Tibet, Ningxia, and Qinghai provinces account for a relatively low proportion of postgraduate pain physicians. See Table 3 for details.

**Table 3:** Construction of pain discipline in China

Province name	D01 Pain department coverage	D02 Pain physicians per million population	D04 current composition of the educational background of pain physicians
Shanghai	39.7	9.2	52.0
Beijing	36.0	15.7	37.8
Guangxi	33.7	11.0	28.2
Qinghai	28.2	19.1	5.2
Jilin	28.0	13.5	18.9
Shaanxi	27.2	15.6	16.8
Hunan	25.4	10.0	17.3
Fujian	24.5	6.9	23.5
Jiangxi	24.3	6.4	19.4
Hubei	23.1	7.2	31.6
Chongqing	21.9	14.0	19.6
Zhejiang	21.3	6.6	22.9
Xinjiang	20.5	10.2	22.1
Anhui	20.4	5.7	17.1
Henan	20.3	5.6	11.8
Tianjin	19.8	9.4	28.8
Gansu	18.9	8.3	14.6
Heilongjiang	17.1	7.2	16.6
Hainan	17.0	6.8	22.2
Jiangsu	16.4	5.4	33.0
Shandong	16.4	6.5	27.7
Guangdong	15.3	4.0	33.2
Hebei	14.9	5.4	13.7
Ningxia	14.7	7.4	3.9
Yunnan	14.6	6.8	11.3
Shanxi	14.5	7.5	20.8
Liaoning	11.0	7.8	36.7
Guizhou	10.9	6.7	17.1
Sichuan	9.4	4.0	20.2
Tibet	7.6	3.8	0.0
Inner Mongolia	5.3	2.4	36.7

## Discussion

This study is the first comprehensive index for chronic pain, reflecting the level of pain health in China and the provincial level. The development of CPHI can provide baseline data for improving pain health levels, project key areas and directions for scientific prevention and treatment of chronic pain, and provide data support for financial investment decision-making and resource allocation.

China has a large population and a vast territory, and the socio-economic development, environmental and geographical factors, living conditions, lifestyles, and medical care utilization of residents in different regions vary considerably. The results of this study indicate this feature which displays as the variety in the prevalence, treatment, and the establishment of pain disciplines in different provinces. Economically developed provinces generally have higher CPHI scores, while economically underdeveloped areas have lower cores. The current pain diagnosis and treatment situation in economically developed provinces is relatively good, while that in economically underdeveloped areas is relatively poor. In addition, it should be emphasized that the scores and rankings of the provinces in CPHI calculation are relative, which means each province has potential for improvement in each dimension or indicator, especially the dimensions/indicators with lower scores or lower rankings. The dimensions or indicators should be paid more attention to find a focus on the progress of pain prevention and treatment in the future.

The results of this study show that the three provinces with severe headache prevalence in China are Fujian, Heilongjiang, and Shanghai. This finding is similar to the results of Yao et al. [10]. The difference in the level of headache prevalence did not reflect the apparent difference in economic level and geographic location. The results of headache epidemiological studies in many countries suggest that the prevalence of headaches is not directly related to the level of economic development. Some studies also indicate that urban–rural or income diversity is weakly related to headache prevalence, and the findings are inconsistent [11]. In resource-rich or resource-deficient countries and regions, headaches have become a health problem worthy of attention [12].

Skeletal muscle pain is the most common of various chronic pains. Compared with other chronic non-communicable diseases, skeletal muscle pain causes the most severe labor loss, absence from work, early retirement, and lower economic income [13, 14]. The prevalence of skeletal muscle pain in Chongqing, Guizhou, and Sichuan provinces is relatively severe. These three provinces are located in southwestern China. This result is similar to the findings of other studies in China [15, 16]. The reason for the regional difference may be because there are many mountainous areas in southwestern provinces, and daily climbing activities increase the burden on bones and joints. In addition, the socio-economic development level of Chongqing, Guizhou, and Sichuan is not high, the proportion of urbanization is low, and the long-term heavy manual labor of rural residents has also led to skeletal muscle damage to a certain extent [16]. The results of the WHO study also suggest that the prevalence of arthritis (the most common type of skeletal muscle pain) among low- and middle-income people is more serious [17].

In China, the outpatient visiting rate for pain patients is 68.2%, the treatment rate is 77.8%, and the satisfaction rate for using analgesics is 64.3%. The results of the study by Zheng et al. showed that 24.1% of Chinese pain patients have never been to the hospital and 36.8% have never been treated for pain [4], which is similar to the results of the CPHI study. This suggests that Chinese people have deficient awareness and management awareness of chronic pain. Our research shows significant differences in the medical consultation rate, treatment rate, and satisfaction of analgesic drug use among Chinese pain patients in different provinces. Therefore, interventions to prevent and reduce chronic pain should be generally implemented in China, addressing modifiable risk factors (such as lifestyle and behavior), taking into account the characteristics of the local chronic pain epidemic, and carrying out patient-centered care. In the longer term, it is more important to enforce education and strengthen the allocation of health resources for people with lower socio-economic status.

The quality of pain discipline construction is related to the management quality of pain patients [18]. The establishment and development of pain disciplines in China have their characteristics. In July 2007, the Ministry of Health of China issued a policy [19], requesting the addition of a first-level diagnosis and treatment subject “Pain Department,” which is mainly responsible for the diagnosis and treatment of chronic pain. Since then, domestic second-level and higher hospitals have successfully carried out pain diagnosis and treatment work, including establishing pain clinics, pain departments, and pain specialist wards. However, the results of this study show that the coverage of pain departments in secondary and tertiary hospitals is not very high. In addition, the treatment and management of headache and musculoskeletal pain should be an essential part of primary health care, and primary health care institutions should play a more critical role in managing and treating pain patients [20–22]. A team-based and patient-centered chronic pain care model is recommended and consistent with the evidence-based, multimodal strategies advanced by the guidelines in China [23–25].

This study has certain limitations. Compared with the development of other disciplines, the discipline of pain is an emerging field of development, and there are relatively few related studies. In the GBD study, we currently use, although the partner organizations have made great efforts to collect all published and unpublished data, the amount and quality of available data on headaches are still limited, which may affect the accuracy of the estimated burden. In addition, the Tibet and Qinghai data in the NHWS are missing, and quantiles are used to fill in, which may reduce the accuracy of the analysis results. To sum up, provincial data used in this study may not be comprehensive enough and have specific errors. The results should be used with caution. It also prompts that the research on pain disciplines and the collection of related health information in China should be further strengthened to ensure that future research results are complete and accurate.

## Conclusion

In conclusion, the economically developed provinces in China have higher CPHI scores, while economically underdeveloped areas have lower scores. The current pain diagnosis and treatment situation in economically developed provinces is relatively good, while that in economically underdeveloped areas is relatively poor. According to the variations in the prevalence and management of chronic pain among populations in different provinces in China, it is necessary to implement chronic pain intervention measures adapted to local conditions. Based on the findings of this study, we suggest that first, comprehensive measures should be taken for pain prevention and control in China. Comprehensive improvement measures should be taken from different perspectives of discipline construction, pain prevention, and treatment to effectively alleviate the chronic pain disease burden in China. Secondly, considering the current positioning of primary medical and health institutions as the gatekeeper of public health, in addition to secondary and tertiary hospitals, primary health care institutions should also carry out pain management and services from the perspective of the population. Finally, the medical and health resources should be allocated to the provinces with lower CPHI scores, such as the northwest and southwest regions in China.

## Data Availability

Data are available on request from the authors.

## Abbreviations

CPHI: China Pain Health Index
AHP: Analytical hierarchy process
China CDC: Chinese Center for Disease Control and Prevention
GBD China: China’s provincial burden of disease research
NHWS: National Health and Wellness Survey
CMDA: Chinese Medical Doctor Association

## References

[1] Treede R-D, Rief W, Barke A, Aziz Q, Bennett MI, Benoliel R, Cohen M, Evers S, Finnerup NB, First MB (2015). A classification of chronic pain for ICD-11. Pain.

[2] Mills SE, Nicolson KP, Smith BH (2019). Chronic pain: a review of its epidemiology and associated factors in population-based studies. Br J Anaesth.

[3] Jackson T, Thomas S, Stabile V, Han X, Shotwell M, McQueen K (2015). Prevalence of chronic pain in low-income and middle-income countries: a systematic review and meta-analysis. Lancet (London, England).

[4] Yongjun Z, Tingjie Z, Xiaoqiu Y, Zhiying F, Feng Q, Guangke X, Jinfeng L, Fachuan N, Xiaohong J, Yanqing L (2020). A survey of chronic pain in China. Libyan J Med.

[5] Liu W, Luo A, Liu H (2007). Overcoming the barriers in pain control: an update of pain management in China. Eur J Pain Suppl.

[6] Xu T, Jiang Y, Mao F, Zhang W, Miao Y, Liu B, Zhou M, Fan B (2021). Construction of China pain health index. Chin J Pain Med.

[7] Vos T, Lim SS, Abbafati C, Abbas KM, Abbasi M, Abbasifard M, Abbasi-Kangevari M, Abbastabar H, Abd-Allah F, Abdelalim A (2020). Global burden of 369 diseases and injuries in 204 countries and territories, 1990–2019: a systematic analysis for the Global Burden of Disease Study 2019. The Lancet.

[8] Clinical publication. https://www.kantar.com/expertise/health/clinical-publications

[9] Measures for the Membership Development and Management of the Chinese Medical Doctor Association http://www.cmda.net/glzd/11154.jhtml

[10] Yao C, Wang Y, Wang L, Liu Y, Liu J, Qi J, Lin Y, Yin P, Zhou M (2019). Burden of headache disorders in China, 1990–2017: findings from the Global Burden of Disease Study 2017. J Headache Pain.

[11] Stovner LJ, Hagen K, Linde M, Steiner TJ (2022). The global prevalence of headache: an update, with analysis of the influences of methodological factors on prevalence estimates. J Headache Pain.

[12] Stovner LJ, Hagen K (2006). Prevalence, burden, and cost of headache disorders. Curr Opin Neurol.

[13] Schofield DJ, Shrestha RN, Cunich M, Tanton R, Kelly S, Passey ME, Veerman LJ (2015). Lost productive life years caused by chronic conditions in Australians aged 45–64 years, 2010–2030. Med J Aust.

[14] Tsang A, Von Korff M, Lee S, Alonso J, Karam E, Angermeyer MC, Borges GL, Bromet EJ, Demytteneare K, de Girolamo G (2008). Common chronic pain conditions in developed and developing countries: gender and age differences and comorbidity with depression-anxiety disorders. J Pain.

[15] Liu Q, Wang S, Lin J, Zhang Y (2018). The burden for knee osteoarthritis among Chinese elderly: estimates from a nationally representative study. Osteoarthr Cartil.

[16] Tang X, Wang S, Zhan S, Niu J, Tao K, Zhang Y, Lin J (2016). The prevalence of symptomatic knee osteoarthritis in China: results from the China health and retirement longitudinal study. Arthritis Rheumatol (Hoboken, NJ).

[17] Brennan-Olsen SL, Cook S, Leech MT, Bowe SJ, Kowal P, Naidoo N, Ackerman IN, Page RS, Hosking SM, Pasco JA (2017). Prevalence of arthritis according to age, sex and socioeconomic status in six low and middle income countries: analysis of data from the World Health Organization study on global AGEing and adult health (SAGE) Wave 1. BMC Musculoskelet Disord.

[18] Atlas of headache disorders and resources in the world 2011 https://www.who.int/mental_health/management/atlas_headache_disorders/en/

[19] Minsitry of Health China: Notice of the Ministry of Health on the addition of “Pain Department” to the “List of Medical Institutions”. 2007

[20] Briggs AM, Woolf AD, Dreinhöfer K, Homb N, Hoy DG, Kopansky-Giles D, Åkesson K, March L (2018). Reducing the global burden of musculoskeletal conditions. Bull World Health Organ.

[21] Briggs AM, Shiffman J, Shawar YR, Åkesson K, Ali N, Woolf AD (2020). Global health policy in the 21st century: challenges and opportunities to arrest the global disability burden from musculoskeletal health conditions. Best Pract Res Clin Rheumatol.

[22] Steiner TJ, Martelletti P (2007). Aids for management of common headache disorders in primary care. J Headache Pain.

[23] Ji Quan YD, Wang J (2019). Chinese expert consensus on chronic musculoskeletal pain management in elderly patients (2019). Chin J Geriatr Res.

[24] Pain Medicine, Chinese Medical Doctor Association: Expert consensus on pharmacological treatment of chronic musculoskeletal pain (2020 edition). Chin J Pain Med 2018, 24(12):881–886.

[25] Yu Shengyuan WQ (2021). Chinese expert consensus on non-drug prevention and treatment of migraine. Neural Injury Funct Reconstr.

